# A review of Janus kinase inhibitors for the treatment of Covid-19 pneumonia

**DOI:** 10.1186/s41232-022-00253-3

**Published:** 2023-01-09

**Authors:** Yoshiya Tanaka

**Affiliations:** The First Department of Internal Medicine, School of Medicine, University of Occupational and Environmental Health, Japan, Kitakyushu, Japan

**Keywords:** SARS-CoV-2, Covid-19, treatment, JAK, baricitinib, NAK family

## Abstract

**Background:**

In inflamed tissue, immune cells are accumulated, and various intercellular signals are involved in the pathogenesis. Janus kinases (JAKs) are typical tyrosine kinases involved in mediating the signaling of multiple cytokines and growth factors and induce the transcription of molecules related to inflammation or immunity via the transcription factor signal transducers and activators of transcription (STAT). Hence, they have garnered significant interest as a therapeutic target. JAK inhibitors have been evaluated as a major drug for remission induction in the treatment of autoimmune diseases such as rheumatoid arthritis.

**Body:**

Covid-19 infection due to SARS-CoV-2 has caused a pandemic, with approximately 660 million infections and 6.7 million deaths worldwide (January, 2023). The prognosis is poor and the major causes of death are respiratory failure attributed to rapid pneumonia, thromboembolism due to a cytokine storm, and multi-organ failure. As a treatment modality, molecular targeted therapy, such as cytokine-targeting therapy, is attracting attention, in addition to antiviral drugs. Baricitinib, a JAK inhibitor, is used for the treatment of severe pneumonia, in addition to antiviral drugs and glucocorticoids. The mechanism of action of baricitinib includes inhibition of viral receptor-mediated endocytosis, which involves the NF-κB activating kinase (NAK) family, and mediating the anti-cytokine effects via JAK 1/2 inhibition. It improves severe pneumonia and reduces mortality.

**Conclusion:**

Thus, the development of molecular targeted drugs with elucidated pathological mechanisms may aid in controlling Covid-19 infection.

## Introduction

The outbreak of pneumonia in Wuhan, China was reported in December 2019. Since then, Covid-19 infection caused by SARS-CoV-2 has become a pandemic and spread like wildfire all over the world [1]. Subsequently, infection peaks have been repeatedly associated with changes in infection/transmission and antigenicity due to various variants [2–4]. As of August 2022, approximately 660 million individuals have been infected worldwide, and 6.7 million individuals have died of infections. The main causes of death are a respiratory failure due to rapidly progressive pneumonia, thromboembolism due to cytokine storm, multi-organ failure, and septic shock [2–5]. With the advent of vaccines, it has become possible to control not only worsening of Covid-19 infection but also the onset of infection to some extent. However, vaccines are not completely effective, and issues such as attenuation of the effect of vaccines and efficacy against variants pose a significant challenge [6, 7]. Antiviral drugs and neutralizing antibodies have successfully reduced disease worsening and mortality rates in patients with mild to moderate disease [7–9]. Nevertheless, these drugs were not administered at appropriate time in many patients.

In patients with moderate to severe disease, severe pneumonia and cytokine storm attributed to the activation of the immune system worsen the prognosis. For these patients, baricitinib, a Janus kinase (JAK) inhibitor, is used for the treatment of SARS-CoV-2 pneumonia requiring oxygen inhalation, as used with the anti-interleukin (IL)-6 receptor antibodies tocilizumab and sarilumab, therapeutic drugs for rheumatoid arthritis (RA), with antiviral drugs or glucocorticoids [10–12]. In addition to the anti-cytokine effect mediating JAK 1/2 inhibition, baricitinib also inhibits viral receptor-mediated endocytosis, which involves the NF-κB activating kinase (NAK) family [13–15]. This article provides an overview of the mechanism of baricitinib for the treatment of SARS-CoV-2 pneumonia.

### JAK/signal transducers and activators of transcription (STAT) signaling pathway

**Fig. 1:**
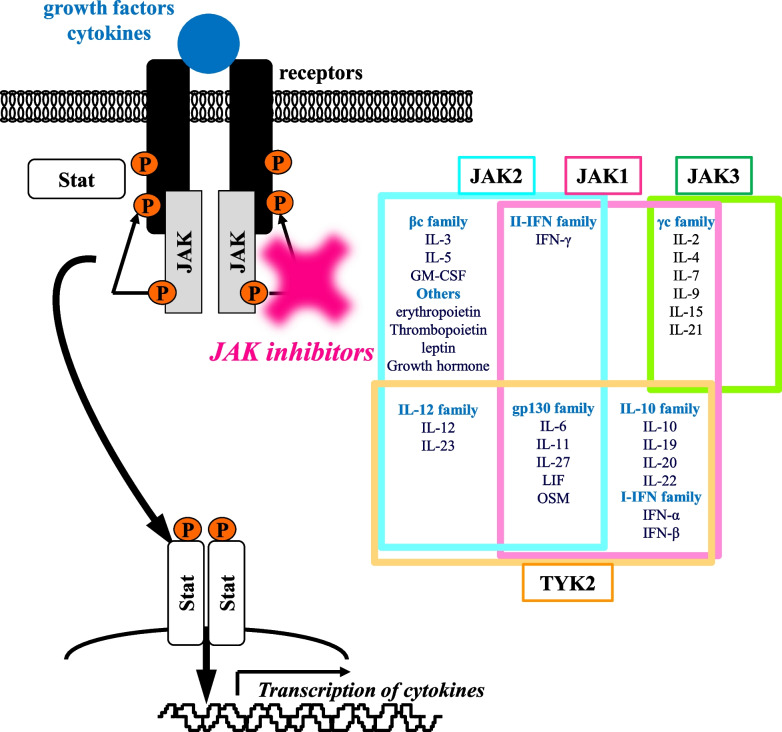
JAK/STAT signal pathway and its inhibitor. Cytokines and growth factors bind to cell surface receptors and activate the associated JAK-STAT signaling pathway

In inflamed tissue associated with autoimmune diseases, immune cells are accumulated, and various intercellular signals are involved in the pathogenesis. JAK is a typical tyrosine kinase involved in the signaling induced by approximately 40 types of cytokines and growth factors [16–19]. Cytokines and growth factors bind to cell surface receptors and activate the associated JAKs, which phosphorylate the intracellular domain of the receptor and bind to the transcription factor STAT. Upon phosphorylation, STAT translocates to the nucleus and mediates the transcription of the multiple genes relevant to inflammation and immunity (Figure 1). Four JAK isoforms, JAK1, JAK2, JAK3, and TYK2, form heterodimers or homodimers to transmit intracellular signals in combination with seven types of STATs and induce transcription of genes encoding various cytokines and cell function-mediating molecules. Therefore, they have garnered significant interest as therapeutic targets for immune diseases.

For the treatment of autoimmune diseases, such as RA and inflammatory bowel disease, low-molecular kinase inhibitors as well as biologics targeting cytokines have been introduced, which revolutionized the therapeutic landscape. JAK3 is activated via stimulation of IL-2, 4, 7, 9, 15, and 21 of the γc family and plays a major role in the immune system. Tofacitinib, which was first approved for the treatment of RA in the USA and Japan in 2012, was developed as a targeted synthetic disease-modifying anti-rheumatic drug (DMARD) with a molecular weight of approximately 312 Da that competitively binds to the ATP binding site of JAK3 and specifically inhibits signaling of these cytokines [20]. It can be orally administered and has a short half-life of approximately 3 hours and pharmacological properties with rapid in vivo metabolism.

### Clinical application of JAK inhibitors for the treatment of RA

The clinical application of JAK inhibitors has been gaining significant attention for the treatment of RA [21]. RA is a systemic autoimmune disease that primarily presents with destructive and persistent polyarthritis. Since joint destruction progresses from the early stage of onset and deformity causes irreversible physical dysfunction, prompt and appropriate diagnosis and treatment are required [22–24]. DMARDs are classified into conventional synthetic DMARD such as methotrexate, targeted synthetic DMARD such as JAK inhibitors, and biological DMARD involving biological agents. These drugs have led to the use of molecular targeted therapy depending on the pathological mechanism. Accordingly, remission is the treatment goal before joint destruction is occurred. If remission is maintained, the progression of structural joint damage and physical dysfunction could be controlled for a long period.

Currently, the following JAK inhibitors are being used for RA: tofacitinib, baricitinib, peficitinib, upadacitinib, and filgotinib [21, 22]. Global phase III clinical studies have revealed that the clinical efficacy of these drugs is significantly higher than placebo, and baricitinib and upadacitinib showed significantly stronger efficacy than adalimumab, an anti-TNF-antibody in patients with methotrexate-resistant RA [25, 26]. Currently, JAK inhibitors can be applied for the treatment of a broad range of immune/inflammatory diseases [21]. Tofacitinib has already been applied to moderate to severe ulcerative colitis, and indications of baricitinib and upadacitinib have been expanded to treat atopic dermatitis. Clinical studies are ongoing for a number of autoimmune diseases, including systemic lupus erythematosus, psoriasis, and Crohn's disease.

However, since biologics completely control a specific molecule and JAK inhibitors block multiple targets, management and treatment for serious side effects are required. The U.S. post-marketing surveillance studies expressed concerns that the risk of cardiovascular disorder, malignant tumors, thrombosis, and death associated with these inhibitor molecules may be higher than that associated with TNF inhibitors. Even though JAK inhibitors are orally administered, they should not be easily prescribed owing to the ease of administration [21]. The use of JAK inhibitors should be limited to specialists who can provide systemic management, and screening before use and monitoring during treatment should be thoroughly performed.

### Covid-19 pneumonia and the treatment

In Covid-19 caused by SARS-CoV-2, latency from exposure to onset is approximately 5 days, while the median duration is less than 3 days for the omicron variant [2–7]. The clinical course and disease worsening vary depending on the vaccination status as well as variants. Approximately 20% of patients develop pneumonia requiring oxygen administration [3–5, 10]. Symptoms such as cold and dry cough first occur, followed by sputum. When respiratory failure progresses, shortness of breath and dyspnea are observed. Chest computerized tomography shows abnormal findings in approximately 90% of patients, within 3 to 5 days after the onset. Multiple ground-glass opacities are observed bilaterally in the lungs from the upper to lower lobes and are characteristically round in shape on the peripheral side of the subpleural region. Approximately 10 days after the onset, disease worsening is observed in approximately 5% of patients, causing acute respiratory distress syndrome (ARDS) and cytokine storm, leading to severe inflammation; 2-3% of cases become fatal. Elevated serum IL-6 and IFN-λ3 levels are considered as prognostic factors. These processes also differ depending on the vaccination status and variants. The mortality rate increases with age. Approximately 30% of individuals aged 80 years or older died before the dissemination of vaccination. Pulmonary embolism and cerebral thromboembolism were the causes of death in many cases, which are triggered by vascular disorder attributed to severe inflammation induced by cytokine storm, as well as respiratory failure and multi-organ failure caused by diffuse alveolar disorder associated with capillary congestion attributed to microthrombi.

Treatment of Covid-19 is broadly divided into (1) antiviral drugs, (2) immunosuppressive drugs, (3) anticoagulants, and (4) other adjuvant therapies [7–12]. The drugs are used according to disease severity and clinical manifestations. When respiratory function deteriorates, respiratory therapies, such as oxygen therapy, mechanical ventilation, and extracorporeal membrane oxygenation (ECMO), are combined. Various criteria are used for severity assessment in each country, including the National Institute of Allergy and Infectious Diseases (NIAID) 8-point ordinal scale and the Japanese Ministry of Health, Labour and Welfare 4-point severity scale [27].

The principle of treatment is to inhibit the proliferation of SARS-CoV-2 and eliminate the virus. Hence, antiviral drugs are used. These drugs are primarily administered to patients with mild to moderate disease because their use in the earlier stages of disease suppresses worsening and death. Remdesivir is an RNA-dependent RNA polymerase inhibitor developed for the treatment of Ebola virus infection [9]. It introduces mutations in the sequence of viral RNA to inhibit viral proliferation. Molnupiravir, a ribonucleoside analog, is also an RNA-dependent RNA polymerase inhibitor used for the treatment of SARS-CoV-2; it also reduces disease worsening. Nirmatrelvir is the primary protease inhibitor of SARS-CoV-2 and is co-administered with ritonavir to delay the metabolism of this drug in the body. When used properly, it is considered to suppress mortality by approximately 90%. Various antiviral drugs are currently under development.

The cocktail therapy involving casirivimab and imdevimab, which are viral neutralizing antibodies against the receptor-binding domain of SARS-CoV-2 spike protein, is administered to patients with mild to moderate disease with a high risk of worsening soon after onset. Significant reduction of viral load and suppression of disease worsening have been demonstrated in clinical studies. Sotrovimab, a monoclonal antibody obtained from patients with SARS, was also shown to reduce disease worsening and death in patients with mild disease shortly after the onset. However, their efficacy may be reduced against variants, and they are recommended for patients who cannot be administered antiviral drugs [11].

In addition to the aforementioned therapies, the use of anticoagulants, such as heparin, is considered in patients with coagulation abnormalities or a history of thrombosis.

However, the efficacy of antiviral agents is limited for advanced pneumonia, ARDS, and severe inflammation due to the cytokine storm. Glucocorticoids have been historically used for severe pneumonia, and dexamethasone is also used for severe pneumonia caused by SARS-CoV-2 infection. A study conducted in the U.K. demonstrated a significant improvement in mortality rates for patients requiring invasive mechanical ventilation. However, no significant difference was observed in mortality among patients with or without ordinary oxygenation. In an analysis of 9,000 US veterans infected with SARS-CoV-2, prognosis was inversely worsened among those requiring oxygenation via nasal cannula. In addition, patients receiving 7.5 mg or more prednisolone equivalent for the treatment of underlying diseases, such as autoimmune connective tissue diseases, carried clear risk factors for increased susceptibility to infection, disease worsening, and poor prognosis [28]. Adverse reactions such as immunosuppression, delay in negative conversion of virus, and hyperglycemia are additional concerns, and hence, the use is controversial.

### Treatment of Covid-19 pneumonia with baricitinib

Treatment needs are not adequately met in patients with moderate to severe respiratory dysfunction. In addition to antiviral therapy intended to suppress viral proliferation in the early stage of infection and control the progression to severe pneumonia, therapeutic strategies are required to control ARDS attributed to acute lung injury and severe inflammation caused by the cytokine storm [10–12]. Covid-19-associated pneumonia, cytokine storm and death associated with them are known to be strongly involved by multiple cytokines such as IL-6, IL-12, IFN-γ and GM-CSF [13]. Baricitinib, a JAK 1/2 inhibitor, exerts a potent anti-inflammatory effect via regulation of multicytokine signals, such as IL-6, IL-12, IFN-γ and GM-CSF, and is expected to inhibit the pathological processes related to SARS-CoV-2 infection. Actually, IL-6 and IFN-γ are risk factors for disease worsening and death associated with Covid-19 pneumonia. Furthermore, since the administration of baricitinib decreases the concentrations of these cytokines in the serum, they are considered to play an important role in pathogenesis [21].

Several reports have shown the usefulness of JAK inhibitors in the treatment of connective tissue disease-associated interstitial lung disease. Anti-MDA5 antibody-positive interstitial lung diseases, which, like Covid-19 pneumonia, have often a rapidly progressive course due to cytokine storms, have been reported regarding the clinical benefit of tofacitinib and baricitinib [29]. It is also reported that in interstitial lung disease associated with systemic sclerosis and rheumatoid arthritis, treatment with baricitinib improved respiratory function and reduced serum fibrosis markers [30]. It is, therefore, suggested that baricitinib has the potential to inhibit fibrosis through inhibition of TGF-β-induced JAK2 phosphorylation [31], in addition to strong inhibition of inflammation by IL-6, IFN-γ and GM-CSF, indicating its usefulness as a therapeutic agent for Covid-19 pneumonia.

**Fig. 2:**
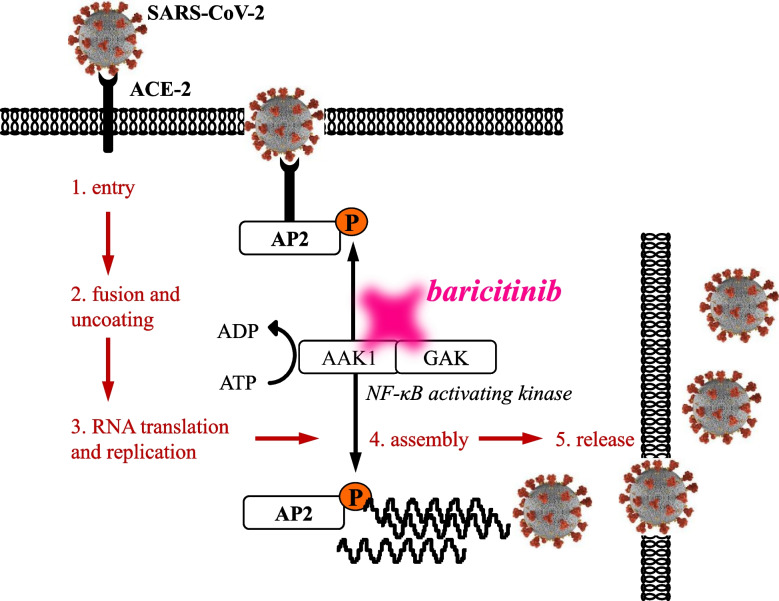
Inhibition of NAK pathway by baricitinib. Baricitinib includes inhibition of viral receptor-mediated endocytosis, which involves the NF-κB activating kinase (NAK) family

The spike protein on the surface of SARS-CoV-2 binds to the angiotensin-converting enzyme (ACE) 2 receptor expressed on the host cell surface, and the host transmembrane protease serine 2 (TMPRSS2) promotes virus invasion into cells. At this point, the virus is incorporated into the cytoplasm via endocytosis mediated by NAK, a signaling molecule, and then proliferates. As an off-target effect, baricitinib may inhibit viral proliferation in cells and transmission of virus between cells via inhibition of the NAK activity. Although mechanisms are unknown, unlike other JAK inhibitors, baricitinib is expected to exert an antiviral effect by NAK inhibition, in addition to the original anti-cytokine effect mediated by JAK inhibition. In vitro studies showed that pretreatment of human hepatocytes with baricitinib significantly reduced the viral load of SARS-CoV-2 in the cytoplasm, but there is currently no clinical evidence to support its efficacy in the early stages of SARS-CoV-2 infection. (Figure 2) [13–15].

**Fig. 3:**
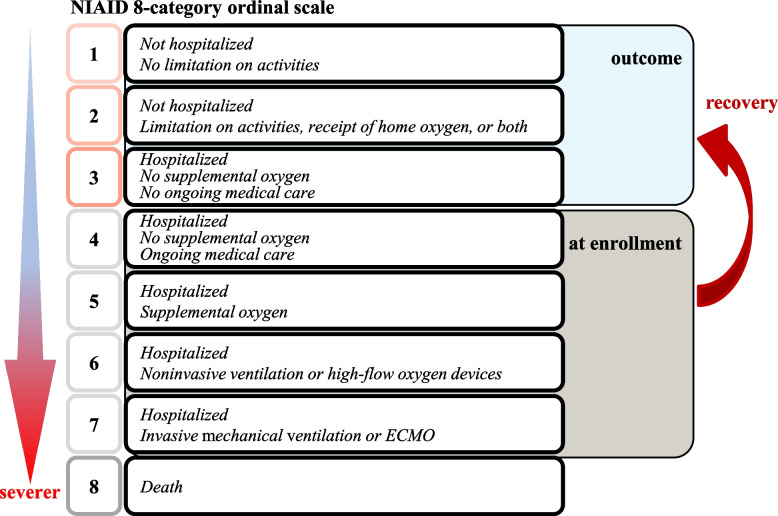
NIAID 8-point ordinal scale was used for the patient inclusion criteria and treatment endpoint in ACCT-2 study. NIAID 8-point ordinal scale was used for the patient inclusion criteria and treatment endpoint. Patients assigned as level 4 (hospitalized but not requiring oxygen supplementation; requiring continuous therapy) to level 7 (hospitalized and managed by invasive mechanical ventilation or ECMO) were included. The primary outcome was clinical improvement to level 1 (not hospitalized or restricting activities) to level 3 (hospitalized but not requiring oxygen supplementation; not requiring continuous therapy) on days 14 by the treatments

In phase III multinational ACTT-2 study conducted among 1,033 hospitalized patients diagnosed with Covid-19, baricitinib or placebo was administered once daily for 14 days or until discharge in addition to remdesivir (within 10 days) [14]. NIAID 8-point ordinal scale was used for the patient inclusion criteria and treatment endpoint (Figure 3). The success of this study was attributed to such clear criteria for implementation and evaluation. Patients assigned as level 4 (hospitalized but not requiring oxygen supplementation; requiring continuous therapy) to level 7 (hospitalized and managed by invasive mechanical ventilation or ECMO) were included. The clinical improvement observed in patients assigned as level 1 (not hospitalized or restricting activities) to level 3 (hospitalized but not requiring oxygen supplementation; not requiring continuous therapy) on days 14 and 28 demonstrated the superiority of the baricitinib and remdesivir treatment compared to the placebo. Thus, this study satisfied the primary endpoint. A significant difference was observed in the odds ratio calculated for the secondary endpoint of clinical improvement based on an ordinal scale on day 14. In a sub-analysis, the odds ratio of improvement based on an ordinal scale on day 14 was significantly higher in the active group for patients assigned as level 5 (hospitalized and requiring supplemental oxygen) to level 7. Patients assigned as level 6 (hospitalized, using non-invasive ventilation or high-flow oxygen device) had the highest odds ratio; however, no significant difference was observed for those assigned as level 4. Accordingly, baricitinib was recommended for patients assigned as level 5 or higher.

In another double-blind study (COV-BARRIER; only 19% was administered remdesivir), baricitinib significantly reduced mortality by 38% on day 28 compared to placebo [32, 33]. The following clinically significant adverse reactions may be observed: infection, gastrointestinal perforation, neutropenia, lymphopenia, decreased hemoglobin levels, hepatic function disorder, jaundice, interstitial pneumonia, and venous thromboembolism. The major route of excretion of baricitinib is the kidney, and decreased clearance is expected to increase baricitinib exposure in patients with renal impairment. Baricitinib is commonly administered at a dose of 4 mg/day. It should be reduced to 2 mg if the estimated glomerular filtration rate (eGFR) is between 30–60 and not recommended if eGFR is ≤ 30. Many kinase inhibitors are currently being examined in clinical studies. In the RECOVERY study involving 10,852 patients with SARS-CoV-2-related pneumonia in the U.K., mortality within 28 days after the onset was significantly lower in the baricitinib group than that observed in the standard care group (12% vs. 14%) [34]. In addition to baricitinib, clinical trials involving several JAK inhibitors without inhibitory effects on NAK activity, including tofacitinib and ruxolitinib, in treating Covid-19 pneumonia are ongoing and should provide valuable information on the usefulness of these agents [35, 36].

**Fig. 4:**
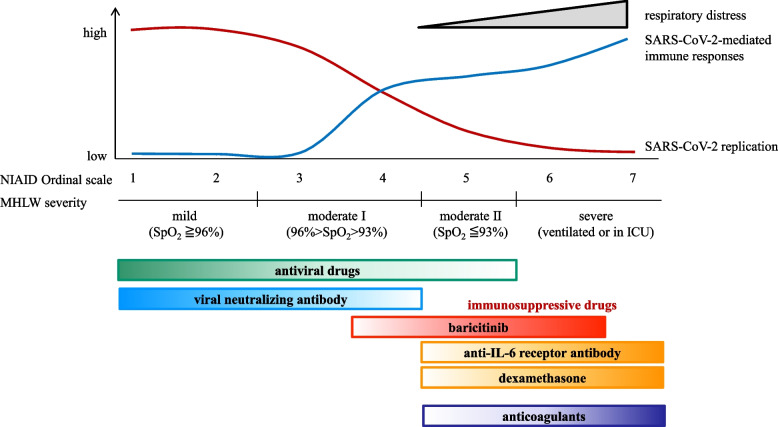
Treatment strategy of Covid-19 based on disease severity. For SARS-CoV-2 infection, it is necessary to select drugs according to the disease severity and pathological condition based on NIAID ordinal scale, severity score by the Ministry of Health, Labor and Welfare, Japan and so on. Antiviral drugs and several immunosuppressive drugs should be differentially selected in each category of the scale

The anti-IL-6 receptor antibodies tocilizumab and sarilumab have also been approved for use in patients with Covid-19 pneumonia requiring oxygen administration by inhibiting the IL-6-mediated cytokine storm related to SARS-CoV-2 infection. The REMAP-CAP study involving 2,046 patients with severe disease demonstrated that tocilizumab and sarilumab were more effective than standard of care and the IL-1 targeting drug anakinra in terms of the survival rate and conversion to ventilator therapy [37]. Furthermore, a meta-analysis conducted by WHO also suggested that combined use with glucocorticoids reduces mortality, and it was recommended that tocilizumab and sarilumab be used in combination with glucocorticoids in hospitalized patients requiring oxygen administration (Figure 4). Antibody therapies against GM-CSF and complement C5a are currently being investigated in clinical studies.

## Conclusion

For SARS-CoV-2 infection, it is necessary to select drugs according to the disease severity and pathological condition. High rate of worsening and mortality was caused by respiratory failure, thromboembolism and multi-organ failure induced by ARDS and cytokine storm. However, with the advent of vaccines, antiviral drugs, and the molecular targeted drugs such as baricitinib and tocilizumab, it has become clear that the risk of disease worsening is markedly reduced and the mortality rate has been also improved, if therapeutic intervention is appropriately applied during the early stages of disease after the onset. Various antiviral drugs and molecular targeted therapies are currently being investigated in clinical studies. However, it would be difficult to suppress the continuous emergence of SARS-CoV-2 variants as long as many individuals are not vaccinated and the most realistic measures taken against Covid-19 are the maintenance of social distance.

## Data Availability

Not applicable

## Abbreviations

ACE: angiotensin-converting enzyme
ARDS: acute respiratory distress syndrome
DMARD: disease-modifying anti-rheumatic drug
ECMO: extracorporeal membrane oxygenation
eGFR: estimated glomerular filtration rate
IL: interleukin
JAK: Janus kinase
NAJK: NF-κB activating kinase
NIAID: National Institute of Allergy and Infectious Diseases
RA: rheumatoid arthritis
STAT: signal transducers and activators of transcription
TNF: tumor necrosis factor

## References

[1] Huang C, Wang Y, Li X, Ren L (2020). Clinical features of patients infected with 2019 novel coronavirus in Wuhan. China. Lancet.

[2] Li Z, Chen Q, Feng L, Rodewald L (2020). Active case finding with case management: the key to tackling the COVID-19 pandemic. Lancet..

[3] Flor LS, Friedman J, Spencer CN (2022). Quantifying the effects of the COVID-19 pandemic on gender equality on health, social, and economic indicators: a comprehensive review of data from March, 2020, to September, 2021. Lancet.

[4] Peeling RW, Heymann DL, Teo YY, Garcia PJ (2022). Diagnostics for COVID-19: moving from pandemic response to control. Lancet..

[5] Zhang Q, Bastard P, Human Genetic Effort COVID, Cobat A, Casanova JL (2022). Human genetic and immunological determinants of critical COVID-19 pneumonia. Nature..

[6] Merad M, Blish CA, Sallusto F, Iwasaki A (2022). The immunology and immunopathology of COVID-19. Science..

[7] Barouch DH (2022). Covid-19 Vaccines - Immunity, Variants, Boosters. N Engl J Med.

[8] Gandhi RT, Lynch JB, Del Rio C (2020). Mild or Moderate Covid-19. N Engl J Med..

[9] Heil EL, Kottilil S (2022). The Goldilocks Time for Remdesivir - Is Any Indication Just Right?. N Engl J Med..

[10] Berlin DA, Gulick RM, Martinez FJ (2020). Severe Covid-19. N Engl J Med..

[11] Rommasi F, Nasiri MJ, Mirsaeidi M (2022). Immunomodulatory agents for COVID-19 treatment: possible mechanism of action and immunopathology features. Mol Cell Biochem..

[12] van de Veerdonk FL, Giamarellos-Bourboulis E (2022). A guide to immunotherapy for COVID-19. Nat Med..

[13] Stebbing J, Krishnan V, de Bono S (2020). Mechanism of baricitinib supports artificial intelligence-predicted testing in COVID-19 patients. EMBO Mol Med.

[14] Kalil AC, Patterson TF, Mehta AK (2021). ACTT-2 Study Group Members. Baricitinib plus Remdesivir for Hospitalized Adults with Covid-19. N Engl J Med..

[15] Goletti D, Cantini F (2021). Baricitinib Therapy in Covid-19 Pneumonia - An Unmet Need Fulfilled. N Engl J Med..

[16] Macchi P (1005). Mutations of Jak-3 gene in patients with autosomal severe combined immune deficiency (SCID). Nature.

[17] O'Shea JJ (2012). JAKs and STATs in immunity, immunodeficiency, and cancer. N Engl J Med.

[18] O'Shea JJ, Kontzias A, Yamaoka K, Tanaka Y, Laurence A (2013). Janus kinase inhibitors in autoimmune diseases. Ann Rheum Dis.

[19] Tanaka Y, Maeshima Y, Yamaoaka K (2012). In vitro and in vivo analysis of a Jak inhibitor in rheumatoid arthritis. Ann Rheum Dis.

[20] van der Heijde D, Tanaka Y, Fleischmann R (2013). Tofacitinib (CP-690,550) in patients with rheumatoid arthritis on methotrexate: 12 month data from a 24 month Phase 3 randomized radiographic study. Arthritis Rheumatol.

[21] Tanaka Y, Luo Y, O’Shea J, Nakayamada S (2022). Janus kinase-targeting therapies in rheumatology: a mechanisms-based approach. Nat Rev Rheumatol.

[22] Tanaka Y (2020). Rheumatoid arthritis. Inflamm Regen..

[23] Smolen JS, Aletaha D, Barton A (2018). Rheumatoid arthritis. Nat Rev Dis Primers.

[24] McInnes IB, Schett G (2017). Pathogenetic insights from the treatment of rheumatoid arthritis. Lancet..

[25] Taylor PC, Keystone EC, van der Heijde D (2017). Baricitinib versus Placebo or Adalimumab in Rheumatoid Arthritis. N Engl J Med..

[26] Smolen JS, Pangan AL, Emery P (2019). Upadacitinib as monotherapy in patients with active rheumatoid arthritis and inadequate response to methotrexate (SELECT-MONOTHERAPY): a randomised, placebo-controlled, double-blind phase 3 study. Lancet.

[27] Beigel JH, Tomashek KM, Dodd LE (2020). Remdesivir for the Treatment of Covid-19 - Final Report. N Engl J Med..

[28] Tam LS, Tanaka Y, Handa R (2021). Updated APLAR consensus statements on care for patients with rheumatic diseases during the COVID-19 pandemic. Int J Rheum Dis.

[29] De Lorenzis E, Natalello G, Gigante L (2020). What can we learn from rapidly progressive interstitial lung disease related to anti-MDA5 dermatomyositis in the management of COVID-19?. Autoimmun Rev.

[30] Tardella M, Di Carlo M, Carotti M (2022). A retrospective study of the efficacy of JAK inhibitors or abatacept on rheumatoid arthritis-interstitial lung disease. Inflammopharmacology..

[31] Wang S, Liu M, Li X (2022). Canonical and noncanonical regulatory roles for JAK2 in the pathogenesis of rheumatoid arthritis-associated interstitial lung disease and idiopathic pulmonary fibrosis. FASEB J.

[32] Marconi VC, Ramanan AV, de Bono S, COV-BARRIER Study Group (2021). Efficacy and safety of baricitinib for the treatment of hospitalised adults with COVID-19 (COV-BARRIER): a randomised, double-blind, parallel-group, placebo-controlled phase 3 trial. Lancet Respir Med.

[33] Ely EW, Ramanan AV, Kartman CE, COV-BARRIER Study Group (2022). Efficacy and safety of baricitinib plus standard of care for the treatment of critically ill hospitalised adults with COVID-19 on invasive mechanical ventilation or extracorporeal membrane oxygenation: an exploratory, randomised, placebo-controlled trial. Lancet Respir Med.

[34] RECOVERY Collaborative Group (2022). Baricitinib in patients admitted to hospital with COVID-19 (RECOVERY): a randomised, controlled, open-label, platform trial and updated meta-analysis. Lancet.

[35] D'Alessio A, Del Poggio P, Bracchi F (2021). Low-dose ruxolitinib plus steroid in severe SARS-CoV-2 pneumonia. Leukemia..

[36] Guimarães PO, Quirk D, Furtado RH (2021). Tofacitinib in Patients Hospitalized with Covid-19 Pneumonia. N Engl J Med.

[37] Investigators REMAP-CAP, Gordon AC, Mouncey PR, Al-Beidh F (2021). Interleukin-6 Receptor Antagonists in Critically Ill Patients with Covid-19. N Engl J Med..

